# The role of metacognition and obsessive-compulsive symptoms in psychosis: an analogue study

**DOI:** 10.1186/s12888-017-1392-1

**Published:** 2017-06-29

**Authors:** Kristen Hagen, Stian Solem, Håvard Berg Opstad, Bjarne Hansen, Roger Hagen

**Affiliations:** 1 Hospital of Molde, OCD-team, Molde, Norway; 2 Haukeland University Hospital, OCD-team, Bergen, Norway; 3 Department of Psychology, Norwegian University of Science and Technology, Trondheim, Norway; 4 St. Olavs University Hospital, Trondheim, Norway; 5 Faculty of Psychology, University of Bergen, Bergen, Norway

**Keywords:** Psychosis, paranoia, hallucinations, OCD, metacognition

## Abstract

**Background:**

Several studies have indicated that obsessive-compulsive disorder (OCD) is a common comorbidity in patients with psychotic disorders, but there is sparse knowledge about the relationship between symptoms of OCD and psychotic symptoms. Metacognitions which guides thinking and coping is theorized to be a transdiagnostic component central for development and maintenance of psychological disorders, OCD and psychosis included. The aim of the study was therefore to explore how symptoms of OCD and metacognitions relate to symptoms of psychosis. Our main hypotheses were that metacognitions would be significantly related to all symptoms of psychological distress, and that there is considerable overlap between symptoms of psychosis and OCD.

**Methods:**

Community controls (*N* = 194) completed an internet survey measuring levels of paranoid ideation, predisposition to hallucinations, symptoms of OCD, depression, anxiety, and metacognitions. Correlations and hierarchical multiple linear regression analyses were used to unveil the relationship between symptoms and beliefs.

**Results:**

Symptoms of OCD showed a strong positive correlation with symptoms of psychosis, and the relationships were still significant after controlling for symptoms of anxiety and depression. Metacognitions also showed strong positive correlations with all symptom measures. Metacognition and OCD-symptoms accounted for 53.8% of the variance in paranoid ideation and 43.8% of predisposition to hallucinations. There was a large overlap between symptoms of psychosis, OCD-symptoms, and metacognitions (30.2–37.3%).

**Conclusions:**

In general, the results suggest considerable overlap between paranoid ideation, predisposition to hallucinations, and OCD and metacognitive beliefs in a non-clinical sample. Further experimental- and clinical studies are needed in order to explore metacognitive models of OCD and psychosis.

## Background

Several studies indicate that obsessive-compulsive disorder (OCD) is a common comorbid disorder for patients with psychotic disorders [1, 2]. In a recent Norwegian study, an OCD prevalence rate of 10.6% was found in patients with first episode psychosis [3]. Still, little is known about the relationship between OCD-symptoms and symptoms of psychosis. Existing research indicates that psychosis with comorbid OCD is associated with a more severe clinical presentation and lower functioning, more depressive symptoms, higher rates of suicidality, although OCD does not seem to affect levels of psychotic symptoms [2–7]. OCD has also been reported to be a risk factor for later development of psychosis [8].

Psychiatric disorders often have overlapping symptoms, which suggests that shared psychopathological processes may be involved in manifestations of symptoms [9]. This also is the case of OCD and psychosis. Both disorders involve intrusions that are difficult to control [10], and differentiating between delusions and obsessions can sometimes be difficult [11]. There is also a considerable overlap between OCD and schizophrenia in terms of course of the disorder and debut age as well as pathophysiological underpinnings, which may explain the increased coexistence of OCD-symptoms and psychotic symptoms [1]. OCD could have a particularly strong connection with schizotypal and psychotic symptoms, as they both are characterized by magical or bizarre thinking patterns [12]. There is also reported a higher than expected prevalence of psychotic-like symptoms, in form of hallucinations, delusions, and/or thought disorder in patients with OCD [13, 14].

Metacognitions have been proposed to be a possible link between psychosis and OCD [15]. Metacognitions refer to beliefs, processes and strategies that monitor, controls or interpret thinking [16]. Metacognitions have been reported to be associated with symptoms of a wide range of psychological disorders, which suggests it may represent a vulnerability to multiple forms of psychopathology [17]. Metacognitive beliefs may also play a role in the development of psychotic symptoms as suggested by the self-regulatory executive function (S-REF) model [18, 19]. This model suggests that vulnerability to psychological dysfunction is linked with a cognitive attentional syndrome (CAS) characterized by heightened self-focused attention, ruminative worry-based processing, and dysfunctional metacognitive beliefs. The S-REF model indicates that as with worry, paranoia may be conceptualized as a strategy more frequently used by people with positive metacognitive beliefs about their paranoia (e.g. thinking of paranoia as a survival strategy). When applying metacognitive principles to paranoia it is theorized that distress may develop when cognitive dissonance is caused by the activation of such positive and negative metacognitive beliefs (e.g. paranoia is uncontrollable) [20]. A similar model of auditory hallucinations has also been developed in which hallucinatory experiences are conceptualized as cognitive intrusions mediated by dysfunctional metacognitive beliefs [21].

In support of the metacognitive model of psychotic symptoms it has been found that dysfunctional metacognitive beliefs are often found in psychotic patients [22], first episode psychosis [23] and psychosis-prone groups [24]. Metacognitive beliefs have been found to be associated with severity of psychotic symptoms [25, 26], although this is not been constantly reported [27]. In a recent meta-analysis [28], it was found that patients with psychosis reported higher scores on all subscales of the Metacognition Questionnaire (MCQ) [29] compared to non-psychiatric controls. It was also found that patients with psychosis reported higher scores on the positive beliefs about worry subscale compared to patients with emotional disorders. Furthermore, higher scores on MCQ seem to be associated both with emotional and psychological disturbance in general, as well as with proneness to psychotic experiences [30], and clinical and non-clinical paranoia [31, 32]. Although these results suggest an association between metacognitions and psychosis, it is important to control for important covariates including anxiety and depression when interpreting these results. A recent meta-analysis provided a comprehensive review of studies that have analyzed the role of metacognitive beliefs in the proneness to hallucinations [33]. The study found that metacognitive beliefs were significantly associated with hallucination-proneness, with effect sizes falling in the moderate-to large range.

In order to further explore the relationship between psychosis and OCD, and metacognition this study set forth to empirically investigate these relationships in a community sample. In testing for associations between OCD and psychosis and predictor variables it is necessary to control for the variance these constructs share with depression and anxiety because any relationship may be spurious and due to overlap with depression/anxiety rather than being specific to symptoms of OCD and psychosis*.* To our knowledge, the influence of metacognitions and OCD on the manifestations of psychotic symptoms has not yet been investigated. This is important, since the functional relationship between OCD and psychosis is not well understood. The metacognitive model has potential transdiagnostic utility, as it emphasizes the importance of metacognitions across psychiatric symptoms. The main hypotheses were therefore that metacognition would be significantly related to all symptoms of psychological distress, and that there is considerable overlap between symptoms of psychosis and OCD.

## Method

### Participants and procedure

A community sample of 194 people took part in an internet survey. They had a mean age of 34.21 (±11.26) and the majority were female (69.6%). The sample consisted of full-time workers (56.7%) and full-time students (24.2%). Some of the participants were receiving disability pensions or were unemployed (7.7%), 1.0% was retirees, and 10.3% were in part-time work or studies. A third (30.9%) of the sample was single. The participants were invited to take part in the survey by invitations on social media. All participants were anonymous, and no renummerations were given.

### Measures

#### Paranoia Scale (PS)

The PS [34] is a 20-item scale that measures subclinical paranoid ideation found in normal individuals. Each item is scored on a 5-point Likert scale, with total scores ranging from 20 to 100. Higher scores reflect higher levels of subclinical paranoia. Subclinical paranoia is defined as a style of thought manifested by exaggerated self-referential biases that arise in ordinary, daily behavior and occurs across a wide range of psychopathologies [34]. The PS was developed for use in analogue samples but has also shown to be useful in persons with paranoid schizophrenia [35]. In the current study the Cronbach’s alpha was 0.93.

#### The launay–slade hallucination scale - revised (LSHS-R)

The LSHS-R [36, 37] is a frequently applied 12-item scale widely used to measure predisposition to hallucinations in healthy individuals. Participants score each item on a 5-point Likert scale (0 = certainly does not apply; 4 = certainly applies). The scores from each item are summed to obtain a total score (0–48), with higher scores indicating a stronger predisposition towards hallucinatory experiences. Cronbach’s alpha in the current study was 0.85.

#### Obsessive-compulsive inventory revised (OCI-R)

The OCI-R is an 18-item self-report questionnaire [38]. OCI-R was developed to examine the presence and severity of obsessive-compulsive symptoms on a five point Likert scale that assess distress associated with various OCD- symptoms from 0 (not at all) to 4 (extremely). The total score of the OCI-R provides information about the OCD severity, but there are also subscales which addresses the severity of the different subtypes of OCD. In this study, the sum of the OCI-R was used as a continuous variable in the statistical analyses, and we the recommended cut-off point of 21 was used to describe clinical symptoms of OCD in the sample [39]. The OCI-R has earlier been shown to be a valid and reliable screening instrument for OCD [38]. A study with a Norwegian sample also found results supporting the validity of OCI-R [40]. In the current study, the OCI-R showed adequate psychometric characteristics with a Cronbach’s alpha of 0.89 for the total score.

#### Patient health questionnaire 9-item (PHQ-9)

The PHQ-9 contains nine items referring to criteria for depression [41] and the answers refer to the past two weeks. The items are scored on a four point Likert scale from 0 (not at all) to 3 (nearly every day). In this study, the sum of scores of the PHQ-9 items was used as a continuous variable for the statistical analysis, and the recommended cut-off point of 10 to describe clinical symptoms of depression in the sample [42]. The PHQ-9 has been shown to have good reliability and validity [42]. Cronbach’s alpha in the current study was 0.86.

#### Generalized anxiety disorder 7-item (GAD-7)

The subjects are asked how often, during the last 2 weeks, they have been bothered by each of the seven core symptoms of generalized anxiety disorder. The items are scored on a four point Likert scale from 0 (not at all) to 3 (nearly every day). In this study the sum of GAD-7 items were used as a continuous variable for the statistical analyses, and the recommended cut-off point of 10 to describe clinical symptoms of anxiety in the sample. The GAD-7 has been shown to have good reliability and validity [43]. Cronbach’s alpha in the current study was 0.87.

#### The metacognition questionnaire (MCQ-30)

The MCQ [44] is a 30-item questionnaire measuring metacognitions, where higher scores indicates more dysfunctional metacognitions. It consists of five subscales: cognitive confidence, negative beliefs (about the danger and uncontrollability of thoughts), beliefs about the need to control thoughts, positive beliefs about worry, and cognitive self-consciousness. Cronbach’s alpha in the current study was 0.93.

### Statistics

To explore the relationship between symptom measures and metacognitions, we used Pearson’s correlations and partial correlational analysis. We also conducted two hierarchical regression analyses; metacognitions and OCD-symptoms were entered in step 1, and age, sex, depressive symptoms and anxiety symptoms were entered in step 2. The dependent variables used in the regressions were PS and LSHS-R. In order to explore which dimensions of metacognitions explained the most variance of psychotic symptoms, we also conducted two multiple forward regressions entering the MCQ subscales as independent variables.

## Results

With regard to symptoms of anxiety, 45.9% of the sample reported no difficulties, 45.9% reported some difficulties, 7.6% reported to have clear difficulties, and 0.5% had extreme difficulties. A total of 11.9% scored above suggested cut-off (10 points) on the GAD-7. With regard to depressive symptoms, 48.4% reported no difficulties, 42.0% reported some difficulties, 8.0% had clear difficulties, and 1.6% reported severe difficulties. On the PHQ-9, 23.9% scored above suggested cut-off of 10 points. With regard to symptoms of OCD, 6.7% scored above suggested cut-off of 21 points on the OCI-R.

For psychotic symptoms, 6.2% scored above suggested cut-off (53 points or more) on the Paranoia scale. Cut-offs scores have been suggested to range from 29 to 32 on LSHS-R and 2.7% scored above 29 and 2.1% scored above 32. A summary of scores on symptoms measures and metacognitive beliefs is presented in Table 1.

**Table 1:** Scores on symptom measures and metacognitive beliefs

	N	Min	Max	Range	Mean	SD
Paranoia scale	194	20	85	20–100	30.62	12.22
LSHS-R	188	0	43	0–48	6.35	7.48
OCI-R	194	0	53	0–72	6.78	7.92
PHQ-9	194	0	23	0–27	5.66	4.63
GAD-7	194	0	21	0–21	5.09	4.10
MCQ	194	30	106	30–120	47.15	13.75
Positive beliefs		6	24	6–24	8.65	2.94
Negative beliefs		6	24	6–24	9.89	3.87
Cogn. confidence		6	22	6–24	9.60	3.70
Need to control		6	23	6–24	8.60	3.55
Cogn. self-consc.		6	24	6–24	10.40	3.64

*LSHS-R* The Launay–Slade Hallucination Scale - Revised, *OCI-R* Obsessive Compulsive Inventory – Revised, *PHQ-9* Patient Health Questionnaire-9, *GAD-7* Generalized Anxiety Disorder-7, *MCQ* Metacognitions Questionnaire-30. Six participants did not answer the LSHS-R, as the participants were able to terminate the study without completing all questionnaires

There were significant gender differences on some of the symptom measures, as women had higher scores than men on LSHS-R, PS, OCI-R and PHQ-9. Women reported higher mean scores on GAD-7 than men (*M*[*SD*] = 5.4 [4.4] vs. 4.3[3.3]), but the difference was not significant (*p* = 0.1).There were no gender differences on MCQ-30. Age showed a weak negative significant correlation with all the symptom measures (correlation coefficients ranging from −0.19 to −0.27).

### The relationship between psychosis, OCD, and metacognitions

Symptoms of OCD were moderately-strongly correlated with symptoms of psychosis. The OCI-R – paranoia partial correlation controlling for anxiety and depression was 0.44 and 0.35 for OCI-R and hallucinations. Metacognitions were significantly correlated with all symptom measures as hypothesized. Correlations between paranoia and subscales of MCQ ranged from 0.43 (cognitive confidence) to 0.62 (need to control thoughts). For LSHS-R and MCQ the correlations ranged from 0.33 (positive beliefs) to 0.63 (need to control thoughts). Negative beliefs and need to control thoughts showed the strongest relationship with all symptom measures. Strength of correlations dropped somewhat when conducting partial correlations controlling for symptoms of depression and anxiety. However, all correlations were still significant except for cognitive confidence and symptoms of paranoia. A summary of the correlations is shown in Table 2.

**Table 2:** Correlations and partial correlations between symptom measures and metacognitive beliefs

	Paranoia	Hallucinations	OCD	Anxiety	Depression
	(PS)	(LSHS-R)	(OCI-R)	(GAD-7)	(PHQ-9)
LSHS-R	0.56 (0.32)				
OCI-R	0.66 (0.44)	0.59 (0.35)			
GAD-7	0.47	0.45	0.54		
PHQ-9	0.61	0.60	0.62	0.69	
MCQ	0.69 (0.50)	0.63 (.41)	0.70 (0.47)	0.61	0.64
Positive beliefs	0.51 (0.38)	0.33 (.15*)	0.49 (0.31)	0.49	0.38
Negative beliefs	0.61 (0.39)	0.53 (.29)	0.64 (0.40)	0.62	0.60
Cogn. Confidence	0.42 (0.14 ns)	0.51 (.27)	0.49 (0.24)	0.36	0.55
Need to control	0.62 (0.41)	0.63 (.44)	0.67 (0.47)	0.51	0.57
Cogn. self-consc.	0.51 (0.39)	0.40 (.24)	0.40 (0.21**)	0.39	0.36

*LSHS-R* The Launay–Slade Hallucination Scale - Revised, *OCI-R* Obsessive Compulsive Inventory – Revised, *PHQ-9* Patient Health Questionnaire-9, *GAD-7* Generalized Anxiety Disorder-7, *MCQ* Metacognitions Questionnaire-30. Partial correlations presented in parentheses control for both anxiety and depression

All correlations otherwise specified were significant (*p* < 0.001)

* *p* < 0.05, ** *p* < 0.01

A hierarchical multiple linear regression was calculated to predict psychotic symptoms (paranoid ideation and predisposition to hallucinations). In the first step, both OCI-R (*β* = 0.339, *t* = 4.92) and MCQ (*β* = 0.455, *t* = 6.60) showed a significant influence on paranoid ideation, *R* ^*2*^ = 0.54, (*F*(2193) = 111.00), *p* < 0.001. In the second step, OCI-R (*β* = 0.273, *t* = 3.80), MCQ (*β* = 0.387, *t* = 5100), and PHQ-9 (*β* = −0.258, *t* = −3,44) showed a significant influence on paranoid ideation, while GAD-7 (*β* = −0.098, *t* = −1.39), age (*β* = −0.034, *t* = −0.68) and sex (*β* = −0.026, *t* = −0.53) were non-significant. Together the second step explained a significant proportion of the variance, *R* ^*2*^ *=* 0.57, (*F*(6187) = 40.61, *p* < 0.001). When using paranoia as the dependent variable; metacognitions and OCD-symptoms explained 53.8% of the variance in symptoms of paranoia. Metacognitions explained 10.6%, and OCD-symptoms explained 5.9% unique variance, while the overlap between paranoid ideation, OCD-symptoms and Metacognitions were 37.3%. A forward regression found that PHQ-9, OCI-R, and the MCQ subscales “positive beliefs” and “cognitive self-consciousness”, were related to paranoid ideation.

Another hierarchical multiple linear regression was also calculated to predict predisposition to hallucinations. In the first step, both OCI-R (*β* = 0.30, *t* = 3.84) and MCQ (*β* = 0.42, *t* = 5.49) showed a significant influence on predisposition to hallucinations *(R* ^*2*^ = 0.43, *F*(2195) = 77.23, *p* < 0.001). In the second step, OCI-R (*β* = 0 .22, *t* = 2.78), MCQ (*β* = 0.34, *t* = 4.09), PHQ-9 (*β* = 0.36, *t* = 4.31), and GAD-7 (*β* = −0.17, *t* = −2.03) showed a significant influence on predisposition to hallucinations, while age (*β* = −0.05, *t* = 0.87) and sex (*β* = −0.05, *t* = 0.89) were non-significant. Anxiety symptoms were positively correlated with predisposition to hallucinations (*r* = 0.45). However, in the regression whilst controlling for OCI-R, PHQ-9, MCQ, gender, and age, the coefficient was negative. This surprise finding was not due to multicollinearity as the VIF (1.1–2.5) and tolerance statistics (0.4–1.0) were within an acceptable range. This indicates that anxiety symptoms are a weaker predictor of predisposition to hallucinations than metacognitions, OCD symptoms, and symptoms of depression. Together the second step explained a significant proportion of the variance *R* ^*2*^ *=* 0.48, *(F*(6181) = 29.22), *p* < 0.001. Using hallucinations as the dependent variable; 43.8% of the variance was explained by metacognitions and OCD. The unique contributions of metacognitions were 9.1%, and OCD-symptoms explained 4.5% unique variance, while there overlap between predisposition to hallucinations, OCD and metacognitions were 30.2%. A forward regression found that PHQ-9, OCI-R and the MCQ subscale “need for control” were related to predisposition to hallucinations. See Table 3 for a summary of the regression analyses.

**Table 3:** Hierarchical linear regression analysis of factors related to psychotic symptoms

Hallucination			Paranoia		
Step 1: (adj. *R* ^*2*^ = 0.43**)	*β*	Sig	Step 1: (adj*. R* ^*2*^ 0.55**)	*β*	sig
OCI-R	0.295	<0.001**	OCI-R	0.339	<0.001**
MCQ	0.422	<0.001**	MCQ	0.455	<0.001**
Step 2: (*R* ^*2*^ Change = 0.054*)			Step 2: (*R* ^*2*^ Change =0.028*)		
OCI-R	0.219	.006**	OCI-R	0.273	<0.001**
MCQ	0.342	<0.001**	MCQ	0.387	<0.001**
Gender	−0.048	0.374	Gender	−0.026	0.599
Age	−0.048	0.388	Age	−0.034	0.496
PHQ-9	0.357	<0.001*	PHQ-9	0.258	<0.001**
GAD-7	−0.165	0.044*	GAD-7	−0.098	0.166
Forward regressions using MCQ subscales					
Gender	−0.052	0.330	Gender	−.016	0.743
Age	−0.048	0.382	Age	−.033	0.511
PHQ-9	0.363	<0.001**	PHQ-9	.339	<0.001**
GAD-7	−0.126	0.109	GAD-7	−.112	0.118
OCI-R	0.202	0.011*	OCI-R	.338	<0.001**
MCQ need to control	0.345	<0.001**	MCQ cogn. Self-consc.	.208	<0.001**
			MCQ positive beliefs	.159	0.013*

* = (*p* < .05). ** *p* < .001

*OCI-R* Obsessive Compulsive Inventory – Revised, *PHQ-9* Patient Health Questionnaire-9, *GAD-7* Generalized Anxiety Disorder-7, *MCQ* Metacognitions Questionnaire-30

## Discussion

The aim of the current study was to explore the role of obsessive-compulsive symptoms and metacognitions in psychotic symptoms. The results suggest considerable overlap between paranoid ideation, predisposition to hallucinations, and OCD and metacognitive beliefs in a non-clinical sample. Correlational analyses indicated strong positive correlations between symptoms of psychosis, metacognitions and OCD-symptoms. For paranoia, all subscales of MCQ (except cognitive confidence) were correlated as well as OCD-symptoms. The same patterns were found in a study of patients with OCD and schizophrenia, were all subscales of MCQ were related to schizophrenia except “cognitive confidence” [15]. The results is also in line with findings from Morrison and colleagues [45] that positive metacognitive beliefs about paranoia are associated with a greater severity of paranoid symptoms.

For predisposition to hallucinations, both OCI-R and all subscales of MCQ were significant correlated. These findings are in line with findings from a recent meta-analysis that indicated that patients with psychosis had significantly higher scores on all the MCQ-subscales compared with non-psychiatric controls, [28]. This is also in line with previous findings of a significant correlation between all subscales of MCQ and LSHS-R in non-clinical sample [46] and in a sample of hallucination-prone subjects [47]. The findings from the study support the S-REF model [18, 19], which, although not targeted specifically at explaining processes in psychosis, predicts that metacognitive beliefs are related to the development and maintenance of emotional disorders [18, 19].

The relationship between predisposition to hallucinations, and metacognitions and OCD-symptoms were still significant after controlling for symptoms of anxiety and depression, as well as sex and age. The regression analysis indicated that metacognitions and OCD-symptoms were significant related to both predisposition to hallucinations and paranoia. In explaining predisposition to hallucinations, a total of 43.8% was explained by metacognition and OCD, and there was a substantial degree of overlap between the three constructs (30.2%). For paranoid ideation, metacognitions and OCD-symptoms accounted for 53.8% of the variance, and the degree of overlap between the constructs were 37.3%. In other words, the results suggest considerable overlap between psychotic symptoms and symptoms of OCD which may be related to metacognitive beliefs. Interestingly, symptoms of anxiety were significant negatively associated with predisposition to hallucinations, in the second step of the regression.

Looking at the subscales of MCQ, the forward regression analysis indicated that cognitive self-consciousness and positive beliefs about worry, as well as OCD-symptoms and depressive symptoms were related to paranoid ideation. Cognitive self-consciousness have been argued to be related to an increased attention to metacognitive beliefs, and thereby may be a trigger of the onset of psychotic symptoms [48]. Patients with psychosis have also been found to report higher rates of positive beliefs about worry, compared to patients at risk for developing psychosis and healthy controls [49]. For predisposition to hallucinations, however, the need to control subscale of MCQ as well as symptoms of OCD and depression were related to predisposition to hallucinations. “Need to control thoughts”, is argued to be central in the metacognitive model as it is activated by worry and rumination and its associated symptoms [50]. In a sample of patients displaying psychotic symptoms, the “need to control thoughts”-subscale was a consistent predictor, and remained significant after controlling for topological characteristics of hallucinations and delusions [51].

As previously mentioned it is important to control for confounding covariates when interpreting the results. We therefore included measures of depression- and anxiety symptoms, but the significant relationship between metacognition and symptoms of psychosis and OCD were still significant after controlling for these variables. Both symptoms of OCD and metacognitions were related to level of psychotic symptoms, after controlling for symptoms of generalized anxiety, depression and demographic factors. These findings indicate that metacognitions may be related to psychotic symptoms as well as other emotional disorders. Future studies need to examine whether the OCD-psychosis relationship is unique as psychotic symptoms could be elevated in other patient groups as well. Overall the results supports previous findings that dysfunctional metacognitive beliefs are associated with both symptoms of psychosis [52], and OCD [53]. The study also indicates that there is a relationship between metacognitions, OCD and psychotic symptoms, and that the concepts have a substantial overlap at subclinical level, even after controlling for anxiety and depression.

### Limitations

Analogue research is commonly used to make inferences about psychological processes in clinical populations and is valuable to research [54]. However, the generalizability of our findings could be compromised by using a non-patient sample and a cross-sectional design. Furthermore, the study is correlational and cannot address causality. Longitudinal studies have, however, indicated that OCD may be a risk factor for later development of psychosis [8]. With respect to metacognitions and OCD, one study found that change in metacognitions accompany improvement in OCD symptoms [53], and treatment that focus on metacognitive mechanisms has led to positive treatment results for OCD [55, 56]. Exposure therapy for OCD is also associated with reductions in psychotic and schizotypal symptoms [57]. With respect to metacognitions and psychosis, a study has indicated that treatment with focus on metacognitions is a feasible for patients with psychosis [58].

## Conclusion

Taken together, these results could be in line with a potential trans-diagnostic utility of the metacognitive model, as it emphasizes the importance of metacognitions across psychiatric symptoms. The results also suggest a considerable overlap between psychotic symptoms (paranoia and hallucinations) and symptoms of OCD, and both of these symptoms were found to be related to metacognitive beliefs. Further experimental- and treatment studies are needed in order to explore the metacognitive model of OCD and psychosis.

## Abbreviations

CAS: Cognitive attentional syndrome
GAD-7: Generalized Anxiety Disorder 7-item
LSHS-R: The Launay–Slade Hallucination Scale – Revised
MCQ-30: The Metacognition Questionnaire
OCD: Obsessive-compulsive disorder
OCI-R: Obsessive-Compulsive Inventory Revised
PHQ-9: Patient Health Questionnaire 9-item
PS: Paranoia Scale
S-REF: Self-regulatory executive function
